# Epigenetic Changes Associated with the Progression of Prion Disease in Syrian Hamsters (Mesocricetus auratus)

**DOI:** 10.21203/rs.3.rs-7850591/v1

**Published:** 2025-10-21

**Authors:** Lexi E. Frank, Nicole Flack, Christopher Faulk, Alyssa J. Block, Jason C. Bartz, Peter A. Larsen

**Affiliations:** University of Minnesota; University of Minnesota; University of Minnesota; Creighton University; Creighton University; University of Minnesota

**Keywords:** chronic wasting disease, CpG Methylation, Gene Expression, Nanopore sequencing, neurodegeneration

## Abstract

Prion diseases are fatal neurodegenerative disorders that affect mammals, including Creutzfeldt-Jakob disease in humans, chronic wasting disease in cervids, and bovine spongiform encephalopathy in cattle. During the disease, abnormally folded prion proteins induce misfolding of normal prion proteins, leading to neurotoxic fibrils and plaques. Epigenetic mechanisms, particularly DNA methylation, are increasingly implicated in prion-like diseases (e.g., Alzheimer’s disease), but their role in prion pathogenesis remains unclear. To investigate, we used nanopore sequencing and RNAseq to measure genome-wide methylation and gene expression in the brains of Syrian hamsters (Mesocricetus auratus) experimentally infected with a hamster-adapted murine synthetic prion strain (n = 9) and age-matched mock-infected controls (n = 9) at 80, 120, and 160 days post-infection (dpi). We identified 1,586, 1,692, and 2,429 differentially methylated regions (DMRs) at 80, 120, and 160 dpi, respectively. Early and mid-stage prion disease (80 and 120 dpi) were skewed toward hypermethylation, whereas late-stage prion disease (160 dpi) was skewed toward hypomethylation. Gene ontology (GO) of nearest genes to DMRs at 160 dpi included terms related to neuron regulation and signaling, neurodevelopment, and cellular stress pathways. We identified 178 differentially expressed genes (DEGs) at 80 dpi, 90 at 120 dpi, and 616 at 160 dpi. The majority of DEGs were downregulated at 80 dpi, and at 120 and 160 dpi, most DEGs were upregulated. Overlap in DEGs across timepoints was limited, and GO terms were related to upregulation of disease/injury response and cell death pathways in later timepoints. Overall, we found stage-specific responses to infection with a transcriptional shift from suppression of immune pathways to widespread immune and inflammation pathway activation. These findings indicate dynamic epigenetic and transcriptional changes marked by progressive and heterogeneous disruption of neuronal structure, function, and communication.

## Introduction

Prion diseases are a group of fatal, transmissible neurodegenerative disorders that impact a variety of mammalian species, including humans, and are transmissible both within and across specific species (*e.g.,* the bovine spongiform encephalopathy epidemic in the United Kingdom) [48, 71]. Prion formation is characterized by global rearrangement of the host-encoded prion protein, PrP^C^, into the infectious self-templating conformation, PrP^Sc^ [14, 34, 47]. Accumulation of prions in neuronal tissues leads to gliosis, neuronal dysfunction that results in the onset of clinical signs of disease and inevitable death of the host. Despite decades of study, effective treatments are lacking, and the molecular mechanisms underlying prion disease pathogenesis are not fully understood [42].

Emerging evidence suggests that epigenetic dysregulation, particularly aberrant DNA methylation, may contribute to prion disease pathology [12, 24, 65]. Epigenetic mechanisms regulate gene expression without altering the nucleotide sequence, playing essential roles in transcriptional regulation and maintenance of genome stability [25, 51, 73]. Disruption of these mechanisms has been linked to various diseases, including many cancers and diseases caused by environmental exposures (*e.g.,* air pollution, heavy metals) [13, 21, 43]. DNA methylation is of particular interest in the context of prion diseases because it plays a well-established role in transcriptional regulation, mediates genome–environment interactions that are important in disease etiology, and — thanks to genome-wide assays such as nanopore long-read methylation calling — can now be interrogated at scale, making it a compelling target for investigating prion disease pathogenesis [37, 44, 55, 65].

Disrupted methylation patterns can both contribute to disease *(e.g.,* Rett Syndrome, cancer) and arise as a consequence of it (*e.g.,* Prader-Willi Syndrome [18, 31]). For example, altered CpG methylation has been implicated in many types of cancer, imprinting disorders, and neurological conditions [11, 21, 37, 61, 68, 74]. In some prion-like neurodegenerative diseases, including Alzheimer’s disease, Parkinson’s disease, and amyotrophic lateral sclerosis, aberrant methylation has been linked to misfolded protein accumulation and neurotoxicity [4, 30, 57, 67]. Although prion and prion-like diseases share mechanistic similarities, prion diseases are generally rarer and less extensively studied [64].

Early epigenetic studies of neurodegenerative diseases reported differences in CpG methylation patterns between controls and patients with Alzheimer’s and Parkinson’s diseases [13, 66]. Similarly, in 2020, researchers found differences between the methylome of sporadic Creutzfeldt-Jakob Disease (sCJD) patients and controls. They identified methylation levels at specific sites associated with prolonged patient survival and methylation signatures with potential for use as a biomarker [12]. A review of epigenomics in prion and prion-like diseases by Hernaiz et al in 2022 found that there are very few studies on the involvement of epigenetic changes in transmissible prion diseases; however, they were able to find 12 common genes with differential methylation compared to controls across studies in scrapie-infected ovine and CJD patient blood [24], suggesting that future studies using consistent methodologies are needed to uncover common genes differentially methylated across all prion-misfolding pathologies and to distinguish how differential methylation contributes to disease.

We are only beginning to understand the various associations between DNA methylation and prion diseases. Most studies of methylation in prion disease have been limited to cross-sectional analyses of non-neuronal tissues in human disease, leaving major gaps in our understanding of epigenetic changes throughout pathological progression in highly affected tissues, such as the brain [12, 66]. Recent advancements in CpG methylation sequencing have provided the tools to address this gap. Oxford Nanopore Technology’s modified base sequencing enables direct detection of CpG methylation at a genome-wide scale, offering a cost-effective alternative to previously used methods, such as bisulfite sequencing [55]. Applying nanopore methylation sequencing technology to established prion disease models, such as *Mesocricetus auratus* (Syrian Hamster), a species that has been used in four decades of prion research [7], allows for longitudinal analysis of methylation changes in neuronal tissues across the course of prion disease.

This study aims to characterize differential methylation and gene expression patterns in Syrian hamster brains over the course of prion disease. Using a dual approach, we utilized single-molecule nanopore sequencing to acquire CpG methylation data and Illumina RNAseq for matched gene expression data to characterize epigenetic and transcriptional landscapes over time. We hypothesized that prion-inoculated hamsters would show an increased number of differentially methylated regions (DMRs) compared to controls throughout the progression of prion disease, and that these regions would be associated with previously characterized gene pathways corresponding to prion disease [16, 17, 19, 29]. Additionally, we hypothesized that gene expression pathways would be altered throughout the course of disease, with changes in the expression of genes relating to neurological disease and injury pathways at later time points.

## Methods

### Ethics statement.

All procedures involving animals were approved by the Creighton University Institutional Animal Care and Use Committee (protocol 1030) and complied with the *Guide for the Care and Use of Laboratory Animals*.

### Prion strains and animal bioassay.

The hamster-adapted murine synthetic prion (HaMSP) strain was generated as previously described (Block et al., 2021). Male Syrian hamsters (Envigo, Indianapolis, IN) were intercranially (i.c.) inoculated with 25 μl of a 1% weight per volume (w/v) brain homogenate in Dulbecco’s phosphate-buffered saline (Mediatech, Herndon, VA) from either uninfected or HaMSP-infected hamsters at the terminal stage of disease. Hamsters were observed three times per week for the onset of clinical signs of prion disease, and the incubation period was calculated as the number of days between inoculation and the onset of clinical signs. Hamsters were individually weighed once per week. Two-tailed Student’s T test (Prism Version 8.4.3, for Mac; GraphPad Software Inc., La Jolla, CA) with a p-value of 0.01 was used to compare incubation periods of disease and animal weights. At selected time points post-infection or at terminal disease, three mock and three HaMSP-infected hamsters were euthanized. All tissues were collected with strain-dedicated tools that are prion decontaminated between animals by immersion in bleach (neat) for 15 minutes at room temperature (RT).

### PrP^Sc^ detection using 96-well immunoassay.

Brain homogenates were digested with 100 μg/ml final concentration of proteinase K (PK) (Roche Diagnostics, Mannheim, Germany) for 1 h at 37 °C with shaking. Detection of PrP^Sc^ using 96 96-well immunoassay was performed as previously described (Kramer and Bartz, 2009). Briefly, the 96-well plate (Millipore, Billerica, MA) was activated with methanol and washed with Tween tris-buffered saline (TTBS) by centrifugation at 470 × g for 30 seconds before use. The PK digested samples were diluted into DPBS to a total volume of 150 μl and loaded onto the activated 96-well plate, centrifuged at 470 × g for 30 seconds, and washed twice with TTBS. The plate was incubated for 20 minutes at RT with 0.3% H_2_O_2_ and then centrifuged at 470 × g for 30 seconds, followed by two TTBS washes. Wells were incubated with 3M guanidine thiocyanate (Sigma Aldrich, St. Louis, MO) for 10 minutes and washed five times with TTBS. The wells were incubated with 5% w/v blotto in TTBS for 30 minutes at 37°C and were next incubated for 1 hour at 37°C with mouse anti-hamster PrP antibody 3F4 (final concentration of 0.1 μg/ml; Chemicon; Billerica, MA). Following five TTBS washes, the wells were incubated with the secondary HRP-conjugated goat anti-mouse antibody for 30 minutes at 37°C (final concentration of 0.1 μg/ml; Thermo Scientific; Rockford, IL.) and washed five times with TTBS. The 96-well plate was developed with Pierce Supersignal West Femto Maximum Sensitivity Substrate according to the manufacturer’s instructions (Pierce, Rockford, IL) and imaged on a Li-Cor Odyssey Fc Imager (Li-Cor, Lincoln, NE). PrP^Sc^ signal intensity was analyzed using Li-Cor Image Studio Software v.1.0.36 (Li-Cor, Lincoln, NE).

### CpG Methylation

#### DNA Extraction and Sequencing

High molecular weight DNA was extracted from 10% homogenized hamster brains (n = 18) in DBPS with the Qiagen MagAttract kit (Qiagen, Hilden, Germany) according to the manufacturer’s protocol. DNA extracts were quantified with a Qubit Fluorometer 4 and the 1X dsDNA High Sensitivity kit (Invitrogen, Carlsbad, California) following the manufacturer’s protocol. An initial assessment of DNA length was performed using gel electrophoresis (1% agarose), and extracts were stored at −20°C until further processing.

DNA was sheared to ~8kb length fragments by passing the total volume through a 28-gauge needle 30 times. AMXPure magnetic beads were used to concentrate the DNA into a 12 μL volume. This volume was used for nanopore library preparation with one of three kits: Native Barcoding kit NBD-SQK114.24, NBD-SQK114.96, or the Ligation Sequencing Kit SQK-LSK114, following the manufacturer’s protocols. Kits differ only in the number of samples that can be included in a library. Briefly, DNA fragments were repaired, and ends were blunted. Then, double-stranded barcodes were ligated onto each sample (this step is not required for the SQK-LSK114 kit). Next, sequencing adapters were ligated onto the DNA. Finally, the libraries were individually loaded onto R.10 PromethION flow cells (FLO-PROM114). Sequencing was performed on the PromethION2 solo device until ~15X coverage of the genome was reached for each sample.

#### Data Analysis

Bioinformatic analysis was performed in R Studio (version 4.4.1) and Ubuntu command line (version 22.04). Basecalling with methylation calling was carried out with the super accuracy model (dna_r10.4.1_e8.2_400bps_supv4.1.0) for 5-Methylcytosine (5mC) and 5-Hydroxymethylcytosine (5hmC). Files were aligned to the NCBI reference *M. auratus* genome (GCF_017639785.1) with Minimap2 (version 2.24) [38]. Aligned BAM files were indexed with Samtools index. Modkit pileup (version 0.3.1) was used to create a bedMethyl file of counts of base modifications for every aligned read. Global methylation of 5mC, 5hmC, and canonical Cytosines was calculated from the bedMethyl files. 5hmC and 5mC modifications were separated into two separate bedMethyl files. MethylKit (1.33.3) was used to identify differentially methylated regions (DMRs) in 1,000 base windows between experimental and control hamsters for each time point [3]. Counts and the mean of hypermethylation and hypomethylation were recorded. Genomation (version 1.36.0) was used to classify DMRs into their nearest feature type (exon, intron, promoter, and intergenic) and calculate significant difference from background with a chi-square test [2]. Given the incomplete annotation status of the *M. auratus* genome, the *Mus musculus* genome annotation (GCF_000001635.27_GRCm39_genomic.gff) was mapped onto the reference *M. auratus* genome (GCF_017639785.1) to provide a more extensive genome annotation using Liftoff (version 1.6.3) [54]. Bedtools closest (version 2.31.1) was used to find the closest gene to a DMR [49]. Hypomethylation and hypermethylation of genes nearest to DMRs were separated into two files per time point, and then each list of DMR-associated genes was provided to the DAVID software (version 2021, knowledgebase 2024q4) and analyzed for Biological Processes enrichment terms [27, 53]. All raw sequence data is available on NCBI’s Sequence Read Archive (SRA) under BioProject number XXX (Numbers to be provided once accepted by a journal). Detailed computational methods used in this workflow are included in the supplementary data (Supplemental Data 1). Additional data is provided within the supplementary information files.

### Gene Expression RNAseq Data

#### RNA Extraction and Sequencing

To preserve RNA quality before isolation, 200 μL of DNA/RNA shield was added to 250 μL of 10% hamster brain homogenate in DBPS per sample, incubated at room temperature for 1 hour to perfuse, and then placed in a −80°C freezer. RNA extraction and RNAseq sequencing were performed at the University of Minnesota Genomics Core. A Qiagen RNeasy kit (Qiagen, Hilden, Germany) was used to extract RNA, following the manufacturer’s instructions. RNA quality and sizing were completed with a Nanodrop spectrophotometer, RiboGreen RNA assay, and the Agilent 2100 Bioanalyzer. 18 unique dual-indexed libraries were created using the Takara/Clontech Stranded Total RNA-Seq Kit v2 - Pico Input Mammalian reagents following the manufacturer’s instructions (Takara, Kusatsu, Shiga, Japan). All libraries were pooled and sequenced on a NovaSeq RNA-seq paired-end 150-bp run to a depth of 40 million reads per sample.

#### Data analysis

Data analysis was performed using R Studio (version 4.4.1) and Ubuntu command line (version 22.04). First, adapters were trimmed using the bbMap script, bbduk (version 35.85) [8]. The *M. musculus* annotation mapped onto the syrian hamster genome (see above) served as the reference. Reads were aligned to this reference using STAR aligner (version 2.7.11b) [15]. FeatureCounts (version 2.0.3) was used to count transcripts [39]. DESeq2 (version 1.44.0) was then used to identify differential expression between treatment and control hamsters and within timepoints 80 dpi, 120 dpi, and 160 dpi with Benjamini-Hochberg adjustment to the p-value [41]. Significantly differentially expressed genes were compared across timepoints, separating positive and negative log2 fold change in expression. DAVID software (version 2021, knowledgebase 2024q4) was used to annotate these sets of genes [27, 53]. All raw sequence data is available on NCBI’s Sequence Read Archive (SRA) under BioProject number XXX (Numbers to be provided once accepted by a journal). Detailed computational methods used in this workflow are included in the supplementary data (Supplemental Data 1). Additional data is provided within the supplementary information files.

## Results

### Pathogenesis of hamsters infected with HaMSP

**Figure 1:**
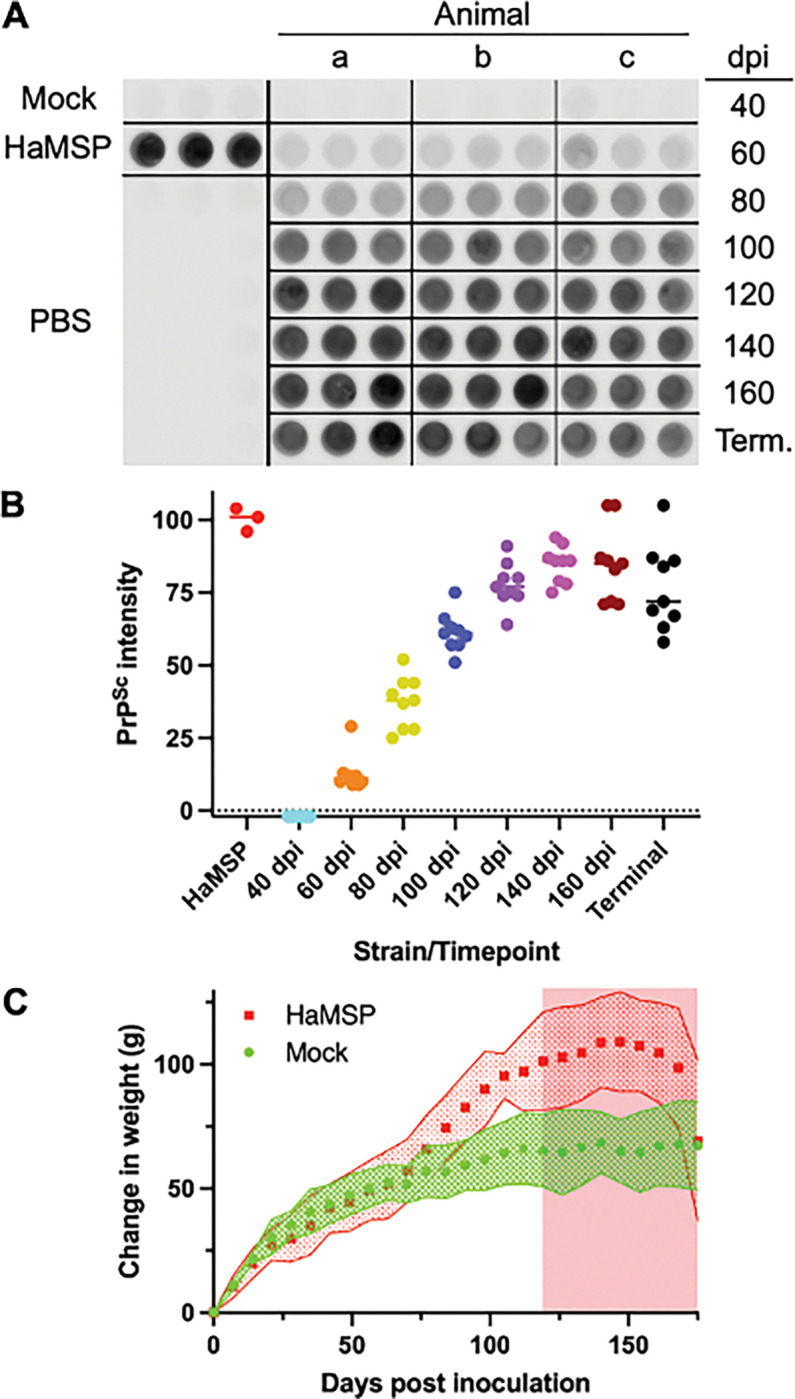
Accumulation of PrP^Sc^ during the time course of hamsters infected with hamster-adapted murine synthetic prions (HaMSP). Dot blot detection (A) and quantification (B) of PrP^Sc^ from hamsters infected with uninfected brain homogenate (Mock) or HaMSP prions at selected time points post infection (dpi) through terminal (Term.) disease. Intensity of PrP^Sc^ accumulation is plotted as a percentage of the PrP^Sc^ accumulation levels in a terminal HaMSP-infected hamster from a previous passage. (C) Compared to mock-infected animals (green circles), HaMSP-infected animals (red squares) significantly (p<0.01) gain weight beginning at 70 days post-infection. The onset of clinical signs of disease occurs at 121±3 days post-infection. The red shaded area indicates the clinical phase of the disease.

Transmission of murine synthetic prions to hamsters resulted in the emergence of a prion strain (HaMSP) characterized by progressive weight gain (Block et al., 2021). We collected 3 mock-infected and 3 HaMSP-infected hamsters every 20 days starting at 40 days post-infection (dpi) until terminal disease by 175 dpi. Detection of PrP^Sc^ from brain homogenates of PK-digested animals revealed the first detection of PrP^Sc^ by 60 dpi that consistently increased until 140 dpi (Figure 1, panels A and B). For these animals, the onset of neurological symptoms of prion disease was first observed 121±3 days post-infection, and a statistically significant (p<0.01) increase in weight of the HaMSP-infected animals compared to age-matched mock-infected controls was first observed at 70 dpi (Figure 1, Panel C). Brain extracts harvested from experimental and control groups at 80, 120, and 160 dpi were used for CpG methylation and gene expression analysis.

### CpG Methylation

**Figure 2:**
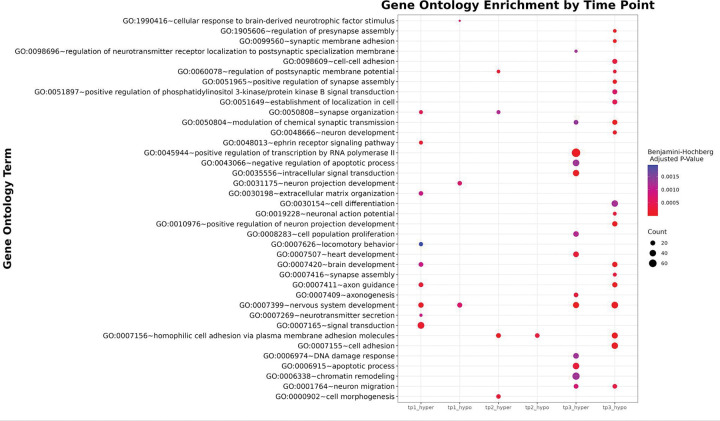
Gene Ontology (GO) term enrichment of differentially methylated genes across time points in prion-infected hamsters. This bubble plot shows significantly enriched GO biological process terms (*Benjamini-Hochberg adjusted p* < 0.002) associated with hypermethylated (_hyper) and hypomethylated (_hypo) genomic regions at three time points (tp1 = 80 dpi, tp2 = 120 dpi, tp3 = 160 dpi). Each bubble represents an enriched GO term at a given time point and methylation direction. Bubble size indicates the number of genes contributing to the enrichment, while color represents the adjusted *p*-value (red = most significant).

DNA extraction concentrations ranged from 41.4 ng/uL to >150 ng/uL. Fragment lengths were approximately 23 Kb before shearing to approximately 8–10 Kb. 15X or higher depth of coverage for the hamster genome was achieved for all samples. Approximately 65 Gigabases per hamster were sequenced for a total of over a terabase of data. Over 22,000,000 CpG sites were captured per hamster. Sequencing statistics (Mean genome coverage, N50, and Mean identity of sequence alignment) can be found in Supplementary Data 2. Global methylation levels were consistent for rodent brain tissues, with a mean of 30.01% of CpGs being canonical cytosine, 60.05% of CpGs being 5-methyl-cytosine, and 9.94% being 5-hydroxy-methyl-cytosine (Supplementary Data 3). Compared to controls, global methylation patterns showed no significant differences or patterns between control and infected hamsters. Regional (1,000 base windows) analysis revealed 1,586 differentially methylated regions (DMRs) at 80 dpi, 1,692 DMRs at 120 dpi, and 2,429 DMRs at 160 dpi. Data at 80 and 120 dpi were skewed slightly toward hypermethylation, and 160 dpi was skewed slightly toward hypomethylation. DMRs were significantly depleted in promoters and enriched in introns at all time points compared to the background (Supplementary Data 4). DMRs were significantly enriched in exons at 120 and 160 dpi compared to the background. Gene Ontology enrichment analysis (Figure 2, Supplemental Data 5) of hypomethylated DMRs showed 56 terms at 160 dpi, 8 terms at 120 dpi, and 7 terms at 80 dpi. Gene Ontology enrichment analysis of hypermethylated DMRs showed 51 significant terms at 160 dpi, 23 terms at 120 dpi, and 30 terms at 80 dpi.

### Gene Expression

**Figure 3:**
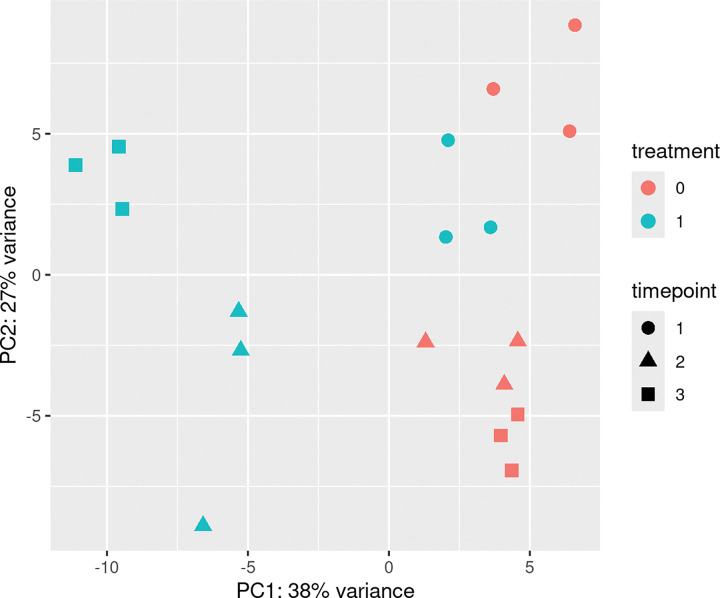
Principal component analysis of RNA-seq gene expression data. Principal component analysis (PCA) was performed to visualize global transcriptomic variation among samples. The first two principal components, PC1 (38% variance explained) and PC2 (27% variance explained), are shown. Each point represents an individual sample, colored according to treatment group (red = control, blue = treatment) and shaped by collection timepoint (circle = timepoint 1, triangle = timepoint 2, square = timepoint 3). Separation along both PC1 and PC2 indicates that variation in gene expression is associated with treatment condition as well as temporal dynamics.

**Figure 4:**
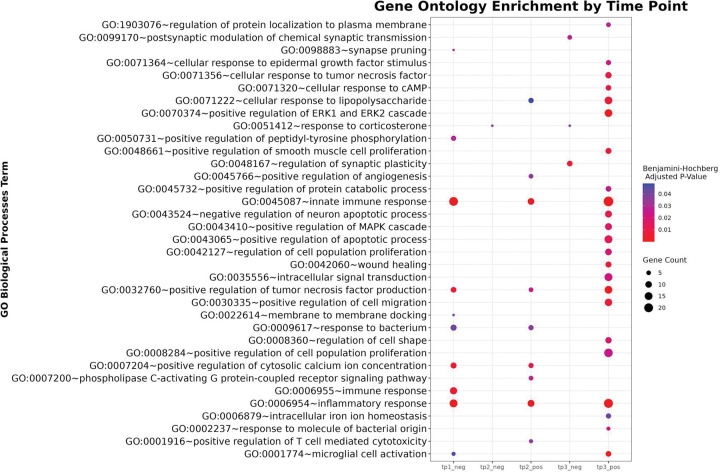
Gene Ontology (GO) term enrichment of differentially expressed genes across time points in prion-infected hamsters. This bubble plot shows significantly enriched GO biological process terms (*Benjamini-Hochberg adjusted p* < 0.05) associated with increased (_pos) and decreased (_neg) gene expression at three time points (80 dpi, 120 dpi, 160 dpi) in prion-infected hamsters. Each bubble represents a GO term enriched at a given time point and expression direction. Bubble size indicates the number of genes contributing to the enrichment, and color reflects the adjusted *p*-value (red = most significant).

RNA concentrations were between 60 and 260 ng/μL and RNA Integrity Number (RIN) scores were between 5 and 8. Over 2,250 million paired-end reads were generated with a mean depth of ≥ 40 M reads per sample. The mean quality scores for all libraries are ≥Q30. A principal component analysis (PCA) revealed that PC1 accounted for 38% of the variance and PC2 accounted for 27% (Figure 3). Separation along both axes indicated that variation in gene expression was associated with both treatment condition and time point. DEGs were skewed toward an increased expression at 120 and 160 dpi and a decrease in expression at 80 dpi. 160 dpi had the most DEGs, for both an increase and a decrease in expression. At 80 dpi, there were 28 unique DEGs with a positive increase in expression and 150 unique DEGs with a decrease in expression. At 120 dpi, there were 67 unique DEGs with a positive increase in expression and 23 unique DEGs with a decrease in expression. At 160 dpi, there were 326 unique DEGs with a positive increase in expression and 290 unique DEGs with a decrease in expression. For DEGs with significant increases in expression compared to controls across time points, there were no DEGs shared by all time points (Supplementary Data 6). For upregulated genes, one DEG was shared between 80 and 160 dpi, 57 DEGs were shared between 120 and 160 dpi, and no DEGs were shared between 80 and 120 dpi. For downregulated genes, there were no DEGs shared by all time points. Zero DEGs were shared between 80 and 160 dpi, seven were shared between 120 and 160 dpi, and no DEGs were shared between 80 and 120 dpi. Gene ontology enrichment analysis (Figure 4, Supplementary Data 5) of DEGs with increased expression showed 23 terms at 160 dpi, nine terms at 120 dpi, and no terms at 80 dpi. Gene Ontology enrichment analysis of DEGs with a decrease in expression showed three significant terms at 160 dpi, one term at 120 dpi, and 10 terms at 80 dpi.

## Discussion

This study provides the first experimental evidence of genome-wide CpG methylation dynamics across the course of a prion infection. By integrating nanopore-based methylation profiling with RNA-seq, we uncovered dynamic changes in DNA methylation and gene expression during prion disease progression. Early phases of infection were characterized by limited immune activation, upregulation of immediate-early stress response and synaptic plasticity genes, and modest DNA methylation changes, whereas late-stage disease exhibited widespread hypermethylation, pronounced neuro-immune and inflammatory responses, synaptic dysfunction, and transcriptional dysregulation.

We identified an increase in DMRs in the experimental group over the three time points. 80 dpi is preclinical, low prion load, while 120 and 160 dpi have reached the critical load of prions sufficient to achieve signs of disease. The development of and change in genomic locations of the DMRs reflect this switch from early to late-stage infection. The enrichment of DMRs in exons in the two later time points indicates stage-specific regulatory changes in gene expression, aberrant or compensatory alternative splicing, or protective or maladaptive transcriptional activity, and may reflect both cell-intrinsic responses to prion pathology and shifts in cell-type composition in affected tissues.

To interpret these findings in greater detail, we next examined how differential methylation and gene expression patterns varied across our prion disease timepoints. Interestingly, we identified distinct signatures that align with both neuronal dysfunction and immune activation as the disease progressed. For example, histone deacetylase 9 (Hdac9), teneurin transmembrane protein 4 (tenm4), and piccolo presynaptic cytomatrix protein (pclo) were within differentially methylated regions across multiple timepoints examined herein. Hdac9 is part of the histone deacetylase family, which regulates chromatin remodeling and gene expression [5, 45, 63, 69] and was the closest transcription start site (TSS) to DMRs at 80 and 160 dpi. The dysregulation of histone deacetylases has been implicated in several neurodegenerative disorders, including Alzheimer’s and Huntington’s diseases, due to their roles in neuronal survival, synaptic plasticity, and inflammation [45, 52, 72]. Tenm4 is involved in axon guidance and synaptic organization, and has been associated with myelination and oligodendrocyte function, processes that are disrupted in neurodegenerative diseases [23, 26, 75]. Tenm4 was the closest TSS to DMRs at 80 and 120 dpi. Pclo plays a critical role in synaptic vesicle trafficking and neurotransmitter release, and its dysfunction has been linked to impaired synaptic transmission and neurodegeneration [28, 46]. This gene was the closest TSS to DMRs at 120 and 160 dpi. The association of these genes with differentially methylated regions across time points suggests that changes to their methylation during prion disease progression could contribute to the molecular mechanisms underlying synaptic loss, neuroinflammation, or neurotoxicity.

Notably, 160 dpi shows the most widespread GO enrichment, particularly for hypermethylated regions, suggesting dynamic and disease progression-dependent epigenetic dysregulation of genes involved in neuroplasticity, synaptic integrity, and neural survival pathways occurs during prion infection. Enriched GO terms include synapse organization, axon guidance, signal transduction, and neuron projection development, pointing to alterations in neural structure and function during disease. The coordinated enrichment of neural development- and synapse-related GO terms highlights potential mechanisms underlying neurodegeneration and altered brain function in prion disease.

Collectively, the gene expression data showed an increasing number of DEGs from the first time point to the last, with little overlap of affected genes between each time point. This trend reinforces a disease-progression dependent transcriptional landscape. Additionally, an overall trend that can be seen in this data is that DEGs (upregulated or downregulated) at 80 dpi often showed the opposite trend at 120 and 160 dpi. This indicates distinct cellular responses occur between the incubation period and after clinical signs emerge, and irreversible neurodegeneration is taking place. DEGs of particular interest were those that exhibited the most significant changes in expression levels or were shared between time points, highlighting key genes involved in the disease process. Among the DEGs of interest, Early Growth Response 1 (Egr1) showed significant upregulation at 80 dpi, followed by downregulation at 120 and 160 dpi. Egr1, an immediate-early gene involved in cellular stress responses and synaptic function, may play a role in prion-induced neurodegeneration [59]. Erg1 was also recently shown to recruit the DNA demethylase, TET1, to remove methylation marks [60]. Similarly, Early Growth Response 4 (Egr4) also showed significant upregulation at 80 dpi, followed by downregulation at 120 and 160 dpi. Egr4 contributes to synaptic plasticity, cellular stress responses, and neuroprotection [36]. C-X-C motif chemokine ligand 13 (Cxcl13) was downregulated at 80 dpi and upregulated in response to prion infection at 120 and 160 dpi. It is a chemokine involved in immune cell trafficking and promotes immune cell recruitment, which may lead to neuroinflammation [62, 76]. Elevated levels of Cxcl13 have been studied in the context of neuroimmunological diseases, such as multiple sclerosis and ALS [22, 62]. Serine (or cysteine) peptidase inhibitor, clade A, member 3M (Serpina3m), a protease inhibitor, was also downregulated at 80 dpi and upregulated at 120 and 160 dpi. Serpina3m regulates inflammation and may block serine protease, thereby chaperoning prion formation [1, 9, 10, 77]. Jun B proto-oncogene (Junb), a transcription factor, is upregulated at 80 dpi and downregulated at 120 and 160 dpi. This gene is typically upregulated during cellular stress and regulates inflammation, apoptosis, and cell survival pathways [33, 50, 70]. However, its downregulation at later time points may indicate departure from a normal cellular response. Transferrin (Trf) is upregulated at 80 dpi and downregulated at 120 and 160 dpi. Transferrin regulates iron homeostasis, and abnormal iron homeostasis in prion diseases may contribute to oxidative stress and neurodegeneration [32]. Trf downregulation in later time points here suggests a change in typical iron homeostasis. This gene has been shown to have changes in transcription in other studies. For example, Singh et al. 2009 [56] showed a downregulation of Trf in vCJD patients and hypothesized that an accumulation of iron in prion protein aggregates may prevent the activation of appropriate molecular pathways for iron deficiency, thus triggering downregulation of Trf. Lastly, Vimentin (Vim), an intermediate filament protein, is upregulated at 80 dpi and downregulated at 120 and 160 dpi. The gene is typically upregulated in glial cells and plays a role in neuroinflammation and the aggregation of misfolded proteins [35].

GO analysis revealed enrichment of the most biological process terms at the final time point (160 dpi), specifically for upregulated genes. The most significant term with an increase in expression at this time point was positive regulation of tumor necrosis factor production (TNF), which refers to genes involved in stimulating the production of TNF molecules. TNF is a chemical messenger that plays a key role in inflammation and immune responses, regulated by various factors, including immune cell activation, inflammatory stimuli, and genetic factors [40]. Protective and detrimental functions of TNF have been noted in ALS patients [20]. At 80 dpi, there are no significant GO terms with an increase in expression; however, GO terms with a decrease in expression at this time point were related to immune response, suggesting that there is little immune response during the incubation period [6, 29, 58]. In contrast, at the next time points (120 and 160 dpi), we see an increase in many of the GO terms that were decreased earlier in disease progression, such as inflammatory and innate immune responses.

The data reveal dynamic, stage-specific transcriptional responses to prion infection. At 160 dpi, the most extensive GO term enrichment is observed, particularly in upregulated genes. Immune and inflammatory processes are prominently enriched, including innate immune response, inflammatory response, microglial cell activation, response to bacterium, and positive regulation of T cell-mediated cytotoxicity, indicating heightened neuroimmune activity during late-stage disease. Concurrently, signaling and stress-response pathways, such as the positive regulation of the MAPK cascade, the ERK1 and ERK2 cascade, tumor necrosis factor production, and phospholipase C-activating G protein-coupled receptor signaling, are significantly enriched, suggesting an upregulation of cell communication and survival mechanisms. In contrast, downregulated genes at 80 dpi are enriched for terms related to immunity, including inflammatory response and innate immune response. At 120 dpi, we start to see the upregulation of immune response and stress-related terms, indicating a transitional phase. Overall, these patterns indicate progressive disruption of neuronal structure and function alongside an escalating immune and inflammatory response, consistent with prion-induced neurodegeneration.

This study provides evidence that CpG methylation and gene expression are altered throughout the incubation period and clinical phase of prion disease in a way relevant to human and animal disease pathogenesis. We provide regions, specific genes, and biological processes associated with methylation and gene expression changes during prion disease, both aligning with previous studies and representing unstudied pathways during prion disease. Further investigation of these genes and pathways is warranted to elucidate the mechanisms underlying prion disease pathogenesis.

## Supplementary Material

Supplementary Files

This is a list of supplementary files associated with this preprint. Click to download.

• supplementarydata1.html

• supplementarydata5GO.xlsx

• supplementarydata.docx
